# Three-Component Accelerometer Based on Distributed Optical Fiber Sensing

**DOI:** 10.3390/s25040997

**Published:** 2025-02-07

**Authors:** Zongxiao Zhang, Qingwen Liu, Rongrong Niu, Zuyuan He

**Affiliations:** State Key Laboratory of Advanced Optical Communication Systems and Networks, Shanghai Jiao Tong University, Shanghai 200240, China; zhangzongxiao@sjtu.edu.cn (Z.Z.); nrr520@sjtu.edu.cn (R.N.); zuyuanhe@sjtu.edu.cn (Z.H.)

**Keywords:** fiber optic sensor, three-dimensional vibration, seismic measurement, accelerometer

## Abstract

The three-component accelerometer array has garnered significant attention in seismic wave detection. In this paper, we designed a three-dimensional optical fiber accelerometer based on a circular cross-section cantilever beam and distributed optical fiber strain interrogator. An externally modulated optical frequency domian reflectometry (OFDR) system with centimeter-level spatial resolution is developed to demodulate the dynamic strain on fiber. An algorithm to reconstruct the three-component acceleration from the strain of the optical fiber was derived, and the factors affecting the errors in reconstruction were also investigated. The developed accelerometer exhibits comparable performance to an electrical accelerometer in the experiment. The correlation coefficient between the reconstructed signal waveforms from the two accelerometers exceeded 0.9, and the angular error was less than 8°. The proposed accelerometer is highly compatible with distributed optical fiber sensing technology, presenting significant potential for long-distance array deployment of three-component seismic wave monitoring.

## 1. Introduction

In recent years, with the rapid development of the oil and gas industry, traditional three-component electronic detectors have encountered challenges such as narrow detection bandwidth, power supply dependencies, and susceptibility to electromagnetic interference [1,2,3]. Optical fiber sensors like distributed fiber optic acoustic sensing (DAS) technology offer advantages including passivity, immunity to electromagnetic interference, resistance to harsh environments such as high temperature and high pressure, ease of deployment, low cost, and long sensing distances. These characteristics have garnered increasing attention from researchers in fields such as seismic wave detection [4,5,6,7].

Common optical fiber accelerometers include interferometric optical fiber sensors [8,9] and fiber Bragg grating (FBG) sensors [10,11]. For vibration sensors based on FBG, researchers have developed sensing structures such as diaphragms [12,13], cantilever beams [14,15,16], and hinges [17,18,19]. Qu et al. designed a low-frequency three-component fiber optic accelerometer based on Fabry–Perot interferometers, consisting of a spherical frame and three-dimensional spring-mass structure, achieving a high acceleration sensitivity of 42.6 dB re rad/g within a working bandwidth of 1–80 Hz [20]. Teng et al. proposed a FBG accelerometer with a double straight-wing structure, which can accurately measure low-frequency vibration signals in the frequency range of 5–22 Hz [21]. However, it did not address the challenge of multi-component sensing. Xiong et al. developed a three-axis vibration sensor with a composite cross-beam elastomer structure. The sensitivities for vibration measurement on the X, Y, and Z axis were 9.7 pm/g, 10.13 pm/g, and 7.45 pm/g, respectively [22]. This design achieves three-component vibration sensing at a single point but faces challenges in long-distance deployment. Qiu et al. proposed an elastic structure composed of cross reeds and cantilever beam, which can measure ultra-low-frequency and weak seismic signals. The minimum detectable frequency is 0.01 Hz and the sensitivity is 606.2 pm/g [23]. This scheme is mainly aimed at low-frequency signals below 20 Hz, and the three-component vibration signal is measured separately by accelerometers in three directions. Zhu et al. designed an FBG vibration sensor with a thin rod cantilever beam to measure vibration signals in the X and Y directions. The frequency measurement range was below 40 Hz and the sensitivity in the X and Y directions was 90.5 pm/g and 102.3 pm/g, respectively [24]. However, this design cannot measure the vibration signal in the Z direction.

With the advancement of DAS technology, researchers have begun exploring multi-component signal acquisition solutions based on DAS. Since DAS can only detect vibration signals in the axial direction of the optical fiber, designing specific sensing models is essential to achieve three-component vibration sensing. Mo et al. developed a three-component fiber optic detector based on DAS, which senses vibration through a spherical mass block, inducing deformation of compliant cylinders in six directions [25]. However, the size of the spherical structure is relatively large. Zhou et al. developed an accelerometer based on FBGs inscribed in three cores of a seven-core fiber, achieving a working frequency bandwidth of 10–220 Hz with a maximum sensitivity of 355 pm/g, and the best azimuthal accuracy of 0.269° [26]. Lim et al. proposed a method using six spirally wound optical fibers to analyze three-component signals [27]. This approach requires obtaining strain signals of six optical fibers on the same cut plane and imposes high demands on spatial resolution.

The existing approach faces two main challenges. The first lies in structural design: using three standalone sensing units to measure the three-component acceleration is not conducive to long-distance deployment or array layouts. It is required to integrate the three-component acceleration sensing capability into a slender structure. The second challenge concerns the performance of the demodulator: when the distributed optical fiber interrogator is adopted for strain measurement, high spatial resolution and high signal-to-noise ratio are critical for accurately reconstructing three-component vibration signals with a slender sensing unit.

In this study, we developed a three-dimensional accelerometer based on a weak FBG array and high-performance optical fiber interrogator. The sensing unit is based on a thin, circular cross-section cantilever beam, enabling deployment in long-distance arrays. This structure can be combined with high-spatial-resolution optical fiber sensing technology to realize long-distance, high-precision measurement of three-component low-frequency vibrations.

## 2. Three-Component Sensing Unit

### 2.1. Structure Design

**Figure 1:**
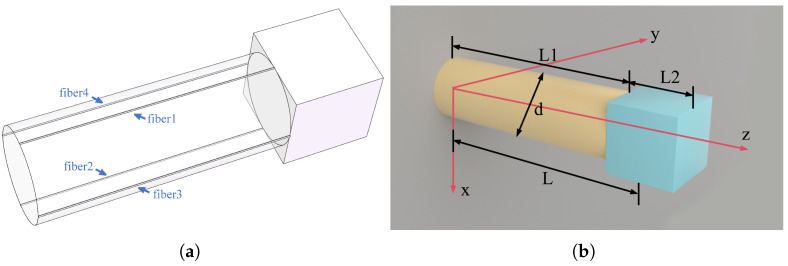
Three-dimensional structure of the accelerometer. (**a**) Schematic of the structure. (**b**) Mechanical model of the sensor unit.

The three-dimensional fiber optic accelerometer we designed features a circular cross-section cantilever beam as the elastic element, and arranges the optical fiber on the surface of the elastic cylinder. By detecting the bending strain and axial strain on the beam, the sensor can reconstruct three-component acceleration and enable three-component vibration sensing. The main components of the fiber optic accelerometer include a fixed base, an elastic cylinder, a mass block, and sensing optical fibers. The overall structure is shown in Figure 1a. Two approaches are used for arranging the optical fibers: the first involves positioning four straight optical fibers along four busbars evenly spaced at 90° intervals on the cylinder’s surface, while the second involves winding the optical fiber helically around the elastic cylinder at a specific spiral angle. Since a single straight optical fiber is sensitive only to strain in one direction within the X-Y plane, multiple straight fibers are required to reconstruct three-component acceleration. In contrast, the spiral winding method theoretically allows a single optical fiber to achieve the same reconstruction.

This structure transforms the detection of three-component acceleration into the measurement of strain along the elastic cylinder. Then, the strain measured by the fiber is converted into the acceleration signals for all three components. This approach enables three-component vibration sensing in slender structures and fulfills the requirements of applications such as three-component seismic wave detection in wells.

### 2.2. Mechanical Model

The mechanical model of a cantilever beam with a circular cross-section is illustrated in Figure 1b. When the inertial mass block experiences an inertial force, the cantilever beam undergoes slight deformation at various points on its surface.This deformation causes the optical fiber bonded to the beam to stretch or compress. In the figure, the height of the elastic cylinder is $L_{1}$, the diameter is *d*, and the side length of the cubic mass block is $L_{2}$. Since the volume of the mass block is non-negligible, the effective length of the beam is given by $L=L_{1}+L_{2}/2$. Compared to traditional thin-walled beams, the mass of the beam must be taken into account. Therefore, in addition to analyzing the strain distribution on the beam’s surface caused by the inertial force of the mass block, it is also necessary to consider the strain induced by the inertial force of the beam itself.

First, we analyze the bending strain distribution on the surface of the elastic cylinder when the sensing unit undergoes vibration in the X and Y directions. The midpoint of the bottom surface of the cylinder is taken as the coordinate origin, with the axial direction of the cylinder defined as the Z axis. Label the mass of the elastic cylinder as $m_{1}$, the density as $\rho_{1}$, the radius as *r*, the cross-sectional area as *A*, Young’s modulus as *E*, the mass of the mass block as $m_{2}$, the density as $\rho_{2}$, and the polar angle on the cross-section as θ. The bending of the cantilever beam primarily results from its moment of inertia, and the inertial force of the beam itself generates additional distributed loads along its length. The bending strain on the surface of the elastic cylinder is expressed as Equation (1):

(1) $$\varepsilon_{bending}=\frac{M_{x}y+M_{y}x}{EI}=-\frac{m_{2}\left(L-z\right)}{EI}\left(a_{x}x+a_{y}y\right)-\frac{\rho_{1}A{\left(L-z\right)}^{2}}{2EI}\left(a_{x}x+a_{y}y\right)$$

Here, $M_{x}$ and $M_{y}$ are the bending moments caused by the acceleration $a_{x}$ and $a_{y}$ in the X and Y directions, respectively, and $I=\frac{\pi d^{4}}{64}$ is the moment of inertia of a circular cross-section.

When the sensing unit is subjected to acceleration in the Z direction, the resulting axial force $F_{z}=ma_{z}+\rho_{1}Aa_{z}L$ will cause the elastic cylindrical cantilever beam to undergo uniform tensile or compressive strain, which can be expressed by Equation (2):

(2) $$\varepsilon_{axial}=\frac{F_{z2}}{AE}=\frac{ma_{z}}{AE}+\frac{\rho_{1}a_{z}L}{E}$$

By combining the above formulas, the total strain on the surface of the elastic cylinder can be expressed by Equation (3), which is the sum of the bending strain and the axial strain:

(3) $$\varepsilon \left(z,\theta \right)=-\left[\frac{64\rho_{2}L_{2}^{3}r\left(L-z\right)}{E\pi d^{4}}+\frac{32\rho_{1}r^{3}{\left(L-z\right)}^{2}}{Ed^{4}}\right]\left(a_{x}\cos\theta +a_{y}\sin\theta \right)+\left(\frac{\rho_{2}L_{2}^{3}}{\pi r^{2}E}+\frac{\rho_{1}L}{E}\right)a_{z}$$

Next, we analyze the natural frequency of the sensing structure. The deflection curve equation at any point along the beam can be derived through the bending moment equation. The maximum deflection $\delta_{max}$ typically occurs at the free end (z=L). The total inertial force $F_{total}$ is the sum of the inertial forces of the mass block and the beam. The equivalent stiffness coefficient of the cylindrical beam $K=\frac{F_{total}}{\delta_{max}}$. Therefore, we can obtain the first-order natural frequency of the sensor unit, as given by Equation (4):

(4) $$f=\frac{1}{2\pi }\sqrt{\frac{K}{m_{total}}}=\frac{1}{2\pi }\sqrt{\frac{24EI}{L^{3}\left(8m_{2}+3\rho_{1}AL\right)}}=\frac{1}{2\pi }\sqrt{\frac{3E\pi d^{4}}{8L^{3}\left(8\rho_{2}L_{2}^{3}+3\rho_{1}\pi r^{2}L\right)}}$$

The phase difference in Rayleigh scattered light or light reflected by weak reflectors over a fiber segment of length $L_{0}$ before and after strain application is $\Delta \phi_{L_{0}}\left(\varepsilon \right)=\frac{\varepsilon L_{0}}{K_{\varepsilon }}$, where $K_{\varepsilon }$ is a constant [5]. Therefore, when the spatial resolution of the demodulation system is B, the sensitivity of the three components of acceleration is given by Equation (5):

(5) $$\begin{array}{cc} S_{x} & =\frac{B}{K_{\varepsilon }}\left[\frac{64\rho_{2}L_{2}^{3}\left(L-z\right)}{E\pi d^{4}}+\frac{32\rho_{1}r^{2}{\left(L-z\right)}^{2}}{Ed^{4}}\right]x\\ S_{y} & =\frac{B}{K_{\varepsilon }}\left[\frac{64\rho_{2}L_{2}^{3}\left(L-z\right)}{E\pi d^{4}}+\frac{32\rho_{1}r^{2}{\left(L-z\right)}^{2}}{Ed^{4}}\right]y\\ S_{z} & =\frac{B}{K_{\varepsilon }}\left(\frac{\rho_{2}L_{2}^{3}}{\pi r^{2}E}+\frac{\rho_{1}L}{E}\right) \end{array}$$

Equation (3) describes the relationship between the strain on the surface of an elastic cylinder and the three components of acceleration. Since optical fibers are only sensitive to strain along their axial direction, in order to reconstruct the three components of acceleration from the strain detected by the fiber, we need to derive the relationship between the strain at each point on the optical fiber and its axial strain based on geometric relationships, as shown in Equation (6) [27]:

(6) $$\varepsilon_{f}=\sin^{2}\left[\left(\alpha -\frac{\pi }{2}\right)\cos\theta \right]\varepsilon_{x}+\sin^{2}\left[\left(\alpha -\frac{\pi }{2}\right)\sin\theta \right]\varepsilon_{y}+\left(\sin^{2}\alpha \right)\varepsilon_{z}$$

Here, $\varepsilon_{f}$ represents the optical fiber axial strain, and $\varepsilon_{x},\;\varepsilon_{y},\;\varepsilon_{z}$ represent the strains along the X, Y, and Z components, respectively. θ is the polar angle, and α is the helical angle of the optical fiber. In the context of seismic wave detection, the strain in the elastic cylinder is small and remains within the elastic range, so $\varepsilon_{x}=\varepsilon_{y}=-\nu \varepsilon_{z}$, where ν is Poisson’s ratio of the material. For each point on the optical fiber, both its polar angle θ and the ordinate *z* are determined, so $\varepsilon_{f}=Ga$, where *G* is the relationship matrix. The corresponding relationship matrix *G* for each segment is then calculated, and the three components of acceleration are reconstructed using the least squares method, as shown in Equation (7):

(7) $$a={\left(G^{T}G\right)}^{-1}G^{T}\varepsilon_{f}$$

### 2.3. Finite Element Analysis

In designing the sensor model, we focus primarily on its natural frequency and sensitivity. Based on the theoretical derivation in Section 2.2, after comprehensive evaluation, we choose nylon as the material of the cylindrical beam, with an elastic modulus of 3.5 GPa, a density of 1.15 $g/{\mathrm{cm}}^{3}$, a diameter of 3 cm, and a length of 8 cm. The stainless steel mass block has an edge length of 3 cm and a density of 7.93 $g/{\mathrm{cm}}^{3}$. Theoretically, the first-order natural frequency of the sensor unit is 225.97 Hz. Assuming a central wavelength of 1550.12 nm for the laser, a refractive index of 1.46 for the optical fiber, and a spatial resolution of 1 cm for the demodulation system, the maximum acceleration sensitivity in the X and Y directions is 2.345 rad/g, and the acceleration sensitivity in the Z direction is 0.107 rad/g.

**Figure 2:**
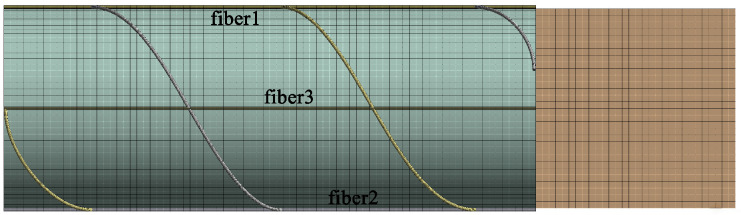
Finite element model after meshing, where both straight fiber and spiral winding fiber are considered.

We perform a finite element simulation analysis of the three-dimensional vibration sensor model using Ansys Workbench. Figure 2 shows the meshed model of the sensor. The vibration modes and resonance frequencies for the first six modes are obtained through modal analysis, as presented in Table 1.

Since high-frequency seismic waves decay rapidly during propagation in the strata, seismic waves primarily consist of low-frequency vibration signals ranging from 10 to 200 Hz. The first-order natural frequency of the sensor unit, obtained through finite element simulation, is 230.08 Hz, which exceeds the frequency range of seismic wave detection and meets the application requirements. Moreover, the first-order natural frequency obtained through simulation closely matches the theoretical value of 225.97 Hz calculated using Equation (4), confirming the accuracy of the theoretical analysis.

**Figure 3:**
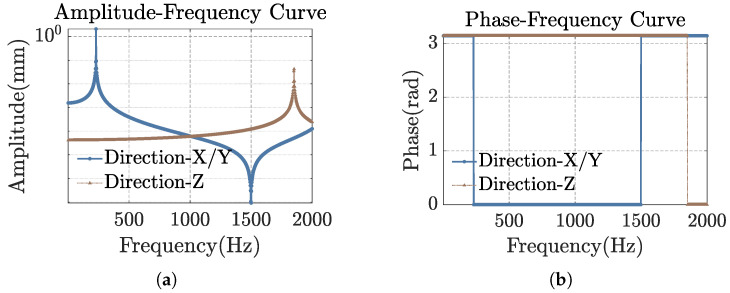
Frequency response of the sensor unit. (**a**) Amplitude–frequency curve. (**b**) Phase–frequency curve.

Next, we conduct a harmonic response analysis on the model. An acceleration of 1 g is applied, and the frequency response of the sensor model is analyzed over the range from 0 to 2000 Hz. The amplitude–frequency and phase–frequency response curves of the sensor unit in all three directions were obtained through finite element simulation, as shown in Figure 3.

The working frequency band of the sensor lies within the flat-amplitude region. As shown in the figure, the amplitude–frequency and phase–frequency characteristic curves of the three directions exhibit abrupt changes at approximately 230 Hz, 230 Hz, and 1850 Hz, corresponding to the first, second, and fourth natural frequencies of the sensing unit. From the flat region in Figure 3a, it can be observed that the operating bandwidth of the sensor is approximately 160 Hz.

**Figure 4:**
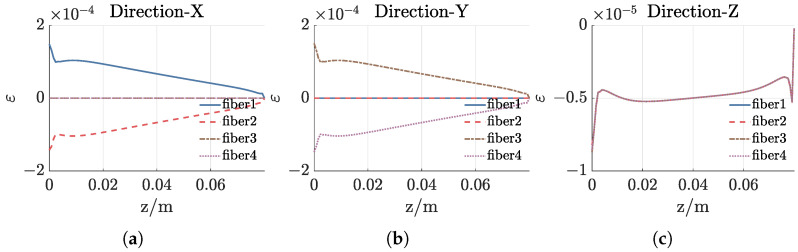
Strain distribution on four straight optical fibers when applying acceleration in three directions. (**a**) Applying acceleration in X direction. (**b**) Applying acceleration in Y direction. (**c**) Applying acceleration in Z direction.

Then, we conduct the static response analysis on the sensor unit. Specifically, an acceleration of 5 g is applied in the X, Y, and Z directions, respectively, to analyze the strain distribution on the four straight optical fibers, as shown in Figure 4. The figure reveals that within the range of 0.02 m ≤z≤ 0.06 m, the strain on the optical fibers varies linearly with the Z coordinate. This linearity arises because the quadratic term in Equation (3) is much smaller than the linear term. Two optical fibers located in the X-Z plane are sensitive to vibrations in the X direction and exhibit opposite magnitudes, while two optical fibers located in the Y-Z plane are sensitive to vibrations in the Y direction and also show opposite magnitudes. When vibrations occur along the Z axis, the strains on all four optical fibers are identical.

**Figure 5:**
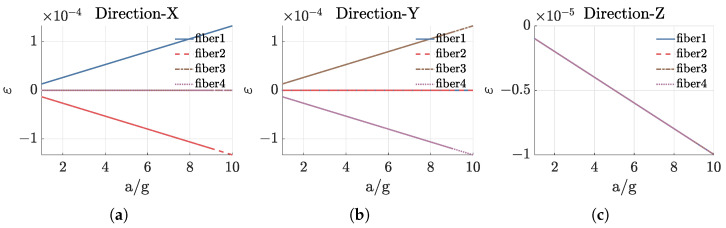
Variation in strain with acceleration when acceleration in three directions is applied. (**a**) Applying acceleration in X direction. (**b**) Applying acceleration in Y direction. (**c**) Applying acceleration in Z direction.

We analyzed the variation in strain with acceleration for the four optical fibers at the cylindrical section located at z = 0.04 m. The acceleration was varied from 1 g to 10 g in increments of 1 g. The results, presented in Figure 5, show that the strain increases linearly with acceleration under loading in each direction. The absolute values of the slopes corresponding to the optical fibers in the sensitive directions are $1.3284\times 10^{-5}$, $1.3284\times 10^{-5}$, and $0.9961\times 10^{-6}$, respectively. These values reflect the sensitivity of the sensing model to three-component acceleration and closely align with the theoretical values obtained by substituting parameters into Equation (3).

**Figure 6:**
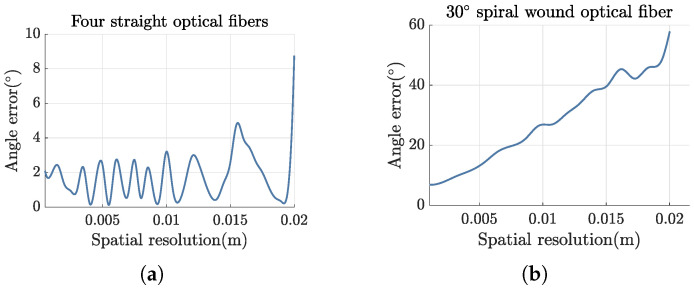
When an oblique acceleration of 1g is applied to the X, Y, and Z components, respectively, the reconstructed angular error changes with the spatial resolution. (**a**) Four straight optical fibers; (**b**) 30° helically wound optical fibers.

Finally, we analyze the reconstruction accuracy of three-component vibration signals for two fiber arrangements: straight fibers and spirally wound fibers. Particular attention is given to the angular error of the three-component reconstruction and the factors influencing this error. Under an applied oblique acceleration of 1 g for the X, Y, and Z components, respectively, the variation in the reconstructed angular error with spatial resolution is examined for four straight fibers and 30° spirally wound fibers, as shown in Figure 6. The figure shows that for straight fibers, the polar angle remains constant, leading to high reconstruction accuracy. When the spatial resolution is within 2 cm, the angular error of the three-component reconstruction is less than 10°. In contrast, for spirally wound fibers, the polar angles vary continuously, and the axial strain along the fibers exhibits non-linear behavior. Consequently, higher spatial resolution is required. To make the spirally wound fiber scheme practical, it is essential to enhance spatial resolution and consider the use of chirped spiral fibers to provide a wider range of winding angle combinations.

## 3. Dynamic Strain Demodulation System

**Figure 7:**
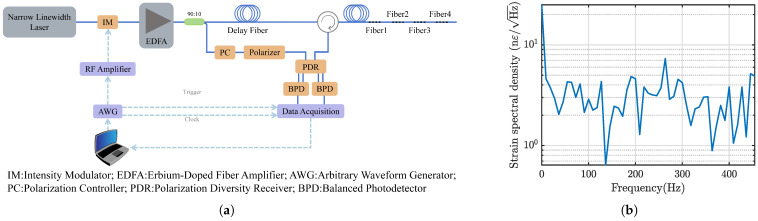
Structure and performance of dynamic strain demodulation system. (**a**) Schematic of external modulation OFDR system. (**b**) Strain spectral density of the last channel of the fiber under test.

For the three-component seismic wave detection based on optical fiber, the signal to be measured is a low-frequency dynamic strain signal. Due to the miniaturization, high precision, and multi-component sensing requirements of the system, high spatial resolution and high signal-to-noise ratio (SNR) are essential. The external modulation OFDR system utilized in this study is illustrated in Figure 7a. The system operates with a sweep duration of 1 ms and a pulse repetition period of 1.1 ms, resulting in a sampling rate of approximately 909.09 Sa/s and a response bandwidth of 454.55 Hz. The sweep frequency range is 16 GHz, giving the system a theoretical maximum spatial resolution of 6.41 mm. The interval of the weak FBG array is 10 mm, and the reflectivity is −40 dB. There are a total of 779 weak FBGs on the sensing fiber. When no vibration signal is applied to the fiber, the strain spectral density of the last channel is shown in Figure 7b, and the strain resolution of the system is 7 $n\varepsilon /\sqrt{\mathrm{Hz}}$.

## 4. Experimental Results

**Figure 8:**
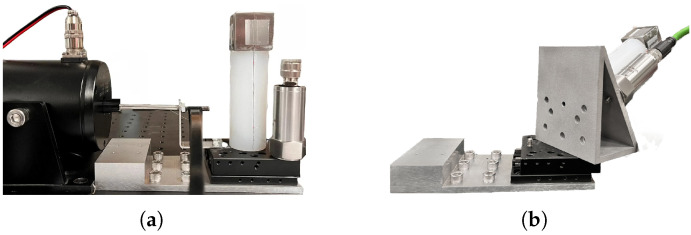
Experimental setup. (**a**) Experimental setup for linearity testing. (**b**) Experimental setup for applying oblique acceleration.

During the experimental preparation phase, four straight optical fibers were affixed to the surface of the elastic cylinder using adhesive, and the cantilever beam was securely attached to the mass block by gluing. A sinusoidal vibration signal was then applied using an exciter, while an triaxial accelerometer (VTall-T163E-A) measured the three-component vibration signal as the reference. The experimental setup is illustrated in Figure 8a.

**Figure 9:**
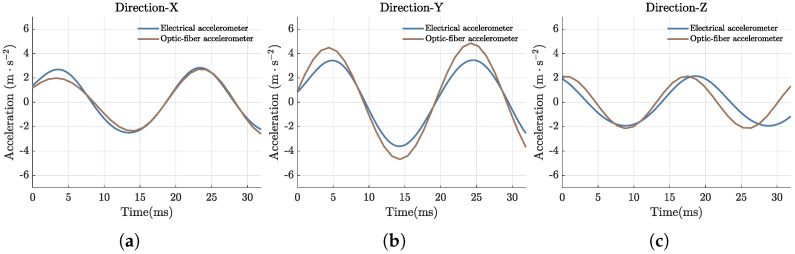
Comparison of acceleration waveforms detected by fiber optic accelerometer and electrical accelerometer when 50 Hz sinusoidal vibration is generated. (**a**) X-axis acceleration; (**b**) Y-axis acceleration; (**c**) Z-axis acceleration.

We used the fiber optic accelerometer designed in this study to detect and reconstruct three-component vibration signals. The fiber optic accelerometer and the electrical accelerometer were mounted on a 60° inclined plane, with the plane oriented at an angle of 57° relative to the vibration direction applied by the exciter. The experimental setup is depicted in Figure 8b. An acceleration signal with a frequency of 50 Hz and an amplitude of 0.5 g was applied using the exciter. Following demodulation, reconstruction, and filtering to remove high-frequency noise outside the measurement range, the waveforms of the three-component acceleration signals were obtained. These waveforms, along with those measured by the reference electrical accelerometer, are shown in Figure 9. As shown in the figure, the reconstructed acceleration signals closely resemble the waveforms measured by the electrical accelerometer. Further calculations show that the angular error of the reconstructed three-component acceleration signal is 7.6°.

**Figure 10:**
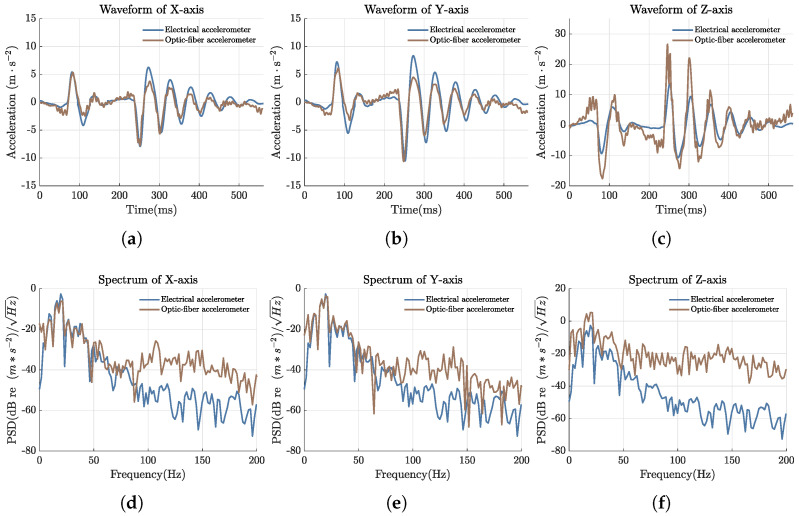
Comparison of signal waveforms and spectra detected by the fiber optic accelerometer and the electrical accelerometer when impulse vibration is generated. (**a**) Waveform of X-axis acceleration. (**b**) Waveform of Y-axis acceleration. (**c**) Waveform of Z-axis acceleration. (**d**) Spectrum of X-axis acceleration. (**e**) Spectrum of Y-axis acceleration. (**f**) Spectrum of Z-axis acceleration.

Then, to validate the detection performance of the fiber optic accelerometer, we applied an impulse vibration as a simulated seismic wave source. After demodulation, reconstruction, and filtering to remove high-frequency noise outside the measurement range, the waveforms and spectra of the three-component vibration signals detected by the fiber optic accelerometer were compared with those measured by the electronic accelerometer. Figure 10a–c depict the acceleration waveforms of the X, Y, and Z components measured by the fiber optic and electronic accelerometers, respectively, while Figure 10d–f show the corresponding signal spectra for the two accelerometers. The figure reveals that the waveforms measured by the fiber optic and electronic accelerometers are consistent across all three axes. The calculated correlation coefficients for the X, Y, and Z directions are 0.9131, 0.9223, and 0.9052, respectively. In the spectrum analysis, the fiber optic accelerometer and the electronic accelerometer demonstrate high consistency at low frequencies. However, due to the absence of a force feedback compensation mechanism, differing response characteristics to external noise, and the installation methods, the spectral consistency between the fiber optic accelerometer and the electronic accelerometer decreases at higher frequencies.

## 5. Conclusions

In this study, we designed and tested a three-component fiber optic accelerometer based on a circular cross-section cantilever beam. This scheme can be combined with distributed optical fiber sensing technology to enable long-distance array deployment and three-component seismic wave monitoring. We have established a mechanical model of the sensor unit and derived the relationship between the strain on the surface of the elastic cylinder and the three-component acceleration. Modal and harmonic response analyses revealed that the first-order natural frequency of the sensor unit was 230.08 Hz. Through static response analysis, we observed that, under constant acceleration, the strain on the straight fiber changes linearly with the Z coordinate within the range 0.02 m ≤z≤ 0.06 m, and at a fixed Z coordinate, the strain is linearly related to the applied acceleration. Sensitivity coefficients were calculated and found to be in good agreement with theoretical predictions. We then analyzed the performance of reconstructing three-component vibration signals for two configurations: straight fibers and spirally wound fibers. Particular attention was given to the effect of the spatial resolution of the demodulation system on angular error during reconstruction. Spiral wound optical fibers require high spatial resolution, while for straight fibers, a spatial resolution within 2 cm ensures an angular error under 10°. Finally, we carried out experimental validations. Sinusoidal and impulse vibration tests were conducted to compare the designed fiber optic accelerometer with a conventional electrical accelerometer. The reconstructed three-component vibration signals demonstrated excellent waveform consistency, with correlation coefficients exceeding 0.9, and an angular error of less than 8°, thereby confirming the sensor system’s effectiveness in accurately restoring three-component vibration signals for low-frequency applications.

Future work will focus on enhancing the sensitivity of Z-axis vibration detection by optimizing the sensor structure and improving the performance of the demodulation system. Additionally, we aim to achieve three-component reconstruction using a single spirally wound fiber, which would provide more significant advantages in long-distance deployment scenarios.

## Figures and Tables

**Table 1:** Resonance frequency of the first six modes of the sensor model.

Order	First	Second	Third	Fourth	Fifth	Sixth
Resonance frequency (Hz)	230.08	230.08	953.2	1851.1	2143.4	2143.4

## Data Availability

Data are contained within the article.
